# Experiences of Public Health Professionals Regarding Crisis Communication During the COVID-19 Pandemic: Systematic Review of Qualitative Studies

**DOI:** 10.2196/66524

**Published:** 2025-03-14

**Authors:** Tsuyoshi Okuhara, Marina Terada, Hiroko Okada, Rie Yokota, Takahiro Kiuchi

**Affiliations:** 1 Department of Health Communication The University of Tokyo Tokyo Japan; 2 Department of Medical Communication Hoshi University Tokyo Japan

**Keywords:** COVID-19, health communication, infodemic, misinformation, social media, SARS-CoV-2, pandemic, infectious, digital age, systematic review, internet, public health, government, health professional, crisis communication, qualitative, disinformation, eHealth, digital health, medical informatics

## Abstract

**Background:**

The COVID-19 pandemic emerged in the digital age and has been called the first “data-driven pandemic” in human history. The global response demonstrated that many countries had failed to effectively prepare for such an event. Learning through experience in a crisis is one way to improve the crisis management process. As the world has returned to normal after the pandemic, questions about crisis management have been raised in several countries and require careful consideration.

**Objective:**

This review aimed to collect and organize public health professionals’ experiences in crisis communication to the public during the COVID-19 pandemic.

**Methods:**

We searched PubMed, MEDLINE, CINAHL, Web of Science, Academic Search Complete, PsycINFO, PsycARTICLES, and Communication Abstracts in February 2024 to locate English-language articles that qualitatively investigated the difficulties and needs experienced by health professionals in their communication activities during the COVID-19 pandemic.

**Results:**

This review included 17 studies. Our analysis identified 7 themes and 20 subthemes. The 7 themes were difficulties in pandemic communication, difficulties caused by the “infodemic,” difficulties in partnerships within or outside of public health, difficulties in community engagement, difficulties in effective communication, burnout among communicators, and the need to train communication specialists and establish a permanent organization specializing in communication.

**Conclusions:**

This review identified the gaps between existing crisis communication guidelines and real-world crisis communication in the digital environment and clarified the difficulties and needs that arose from these gaps. Crisis communication strategies and guidelines should be updated with reference to the themes revealed in this review to effectively respond to subsequent public health crises.

**Trial Registration:**

PROSPERO CRD42024528975; https://www.crd.york.ac.uk/prospero/display_record.php?RecordID=528975

**International Registered Report Identifier (IRRID):**

RR2-10.2196/58040

## Introduction

The COVID-19 pandemic claimed millions of lives. It resulted in a public health crisis and caused economic and social turmoil worldwide. No country, irrespective of region or wealth, was spared the devastating effects of the COVID-19 pandemic. Given that there were no available drugs or vaccines early in the pandemic, communication was an important means of containing the crisis. Even after vaccines were developed, communication to increase trust in the vaccines was central to ending the crisis. Therefore, communication is essential in dealing with a pandemic [1].

Before the COVID-19 outbreak, crisis communication guidelines had been published by the World Health Organization (WHO) [2-4] and crisis communication strategies had been studied by researchers [5-9]. However, when the COVID-19 pandemic started, public health organizations worldwide acknowledged their lack of preparation and training for effective communication during such chaos [10-15]. Furthermore, the communication technology infrastructure has become increasingly complex over the last few decades. Social media platforms now seamlessly connect people to both accurate and false information, which tends to flow to recipients faster than viruses spread [16]. During the COVID-19 pandemic, public health organizations worldwide experienced difficulties with the “infodemic” of misinformation on social media [17]. Before the pandemic, researchers had recognized the importance of management of misinformation and studied countermeasures [18-21]. However, the COVID-19 pandemic highlighted the inexperience of public health agencies in dealing with the influence of misinformation during an emergency [22,23]. Therefore, the COVID-19 pandemic presented public health agencies with unprecedented challenges and highlighted the need to update existing crisis management communication strategies. A crisis is an important opportunity for learning; learning through experience in a crisis is the only way to improve the crisis management process [24,25]. Now that the world has returned to normal following the pandemic, questions requiring reflection have been raised about the crisis management in each country. Therefore, studies are needed to collect and organize data on public health professionals’ experiences in crisis communication worldwide during the COVID-19 pandemic. This work is essential for updating crisis communication strategies to prepare for subsequent public health crises.

We conducted a systematic review of qualitative studies that focused on public health professionals’ experiences in crisis communication during the COVID-19 pandemic in diverse countries. We examined the difficulties that public health professionals experienced during the COVID-19 pandemic, the challenges they faced in overcoming those difficulties, and the needs to be met in future public health crises. We also discussed the gaps between existing crisis communication guidelines and real-world experiences in the COVID-19 pandemic that need to be bridged going forward.

## Methods

### Overview

This systematic review followed the guidelines provided in the PRISMA (Preferred Reporting Items for Systematic Reviews and Meta-Analyses) statement [26]. In addition, we referred to the Sample, Phenomenon of Interest, Design, Evaluation, Research type tool for the synthesis of qualitative evidence [27]. The protocol was previously published [28] and registered with the international Prospective Register of Systematic Reviews (registration: CRD42024528975).

### Literature Search

We searched the following databases on February 7, 2024: PubMed, MEDLINE, CINAHL, Web of Science, Academic Search Complete, PsycINFO, PsycARTICLES, and Communication Abstracts. We filtered our database searches to include articles published from January 1, 2020, to January 31, 2024. We used a combination of keywords with reference to previous studies to search the abstracts in these databases [29-31]: “((government*) OR (ministr*) OR (department*) OR (office*) OR (municipalit*) OR (prefecture*) OR (province*) OR (state*) OR (count*) OR (organization*) OR (institution*) OR (center*) OR (agenc*) OR (sector*) OR (authorit*)) AND ((covid-19) OR (coronavirus) OR (sars-cov-2)) AND ((interview*) OR (focus group*) OR (questionnaire*) OR (survey*)) AND ((communicat*) OR (messag*) OR (inform*) OR (recommend*) OR (announce*)) AND ((qualitative) OR (mix method)).”

### Study Selection

We used Rayyan software (Qatar Computing Research Institute) [32] to screen the identified studies and automatically remove duplicates. Study inclusion and exclusion criteria are shown in Textboxes 1 and 2.

Titles and abstracts were independently screened to identify eligible studies using selection criteria established by the first author (TO) and the second author (MT). Then, the full texts of the remaining studies were screened independently by the first and second authors. Any disagreements during the screening process were discussed until consensus was reached, assisted by the third author (HO), as necessary.

**Study inclusion criteria.**

- The study aim was to investigate public health professionals’ experiences in crisis communication during the COVID-19 pandemic in the digital age with the infodemic of misinformation on social media.
- Regarding study content: qualitative studies of communications from governments and public health agencies to the public focusing on addressing the infodemic of misinformation on social media platforms.
- Regarding design: studies with qualitative data (eg, interviews, documents, and free-text questionnaire responses), those that used content analysis of qualitative data, reviews of qualitative studies, and mixed methods studies with qualitative results that met the study aim.
- Studies on individuals (irrespective of age, gender, ethnicity, or nationality), such as officials, health professionals, and researchers working for governments and public health agencies.
- Gray literature (information produced outside traditional publishing and distribution channels, such as conference proceedings and theses) if sufficient information was provided to confirm its eligibility (ie, full-length descriptions of research objectives, methods, results, discussion, and conclusions).
- Papers written in English and conducted from (and including) January 2020.

**Study exclusion criteria.**

- Quantitative studies with quantitative data (eg, observational and interventional studies)
- Studies on journalists in media companies, patients, and the public
- Studies not published in full-text format
- Non–English-language papers
- Studies that did not meet the study aim that public health professionals’ experiences in crisis communication during the COVID-19 pandemic in the digital age with the infodemic of misinformation on social media (eg, those on content analysis of media information, information searches by the public, COVID-19 patient management in hospitals, and patient-provider communication)

### Quality Assessment

The Joanna Briggs Institute Critical Appraisal Checklist for Qualitative Research was used to assess the methodological quality of eligible studies [33,34]. This Joanna Briggs Institute checklist assesses the descriptive, interpretative, theoretical, and evaluative validity of qualitative studies. The 10 items of the checklist are evaluated as “yes,” “no,” “unclear,” or “not applicable.” The first (TO) and second (MT) authors independently performed quality assessments of the included studies. Any disagreements were discussed until consensus was reached, assisted by the third author (HO) as necessary.

### Data Synthesis

Thematic synthesis was used to synthesize the collected data [35]. Thematic synthesis is recommended as a systematic method for synthesizing qualitative evidence [36]. In the first stage, free line-by-line coding of texts and quotations in the results and discussion sections of the included studies was conducted by TO. Next, 2 reviewers (TO and MT) independently grouped similar codes and generated data-driven descriptive themes. Consensus was reached through discussion, and the third reviewer (HO) was consulted when necessary. Finally, TO developed analytical themes by organizing the descriptive themes generated in the previous stage. This process of developing analytical themes involved repeated discussions among TO, MT, and HO.

## Results

### Study Characteristics

**Figure 1:**
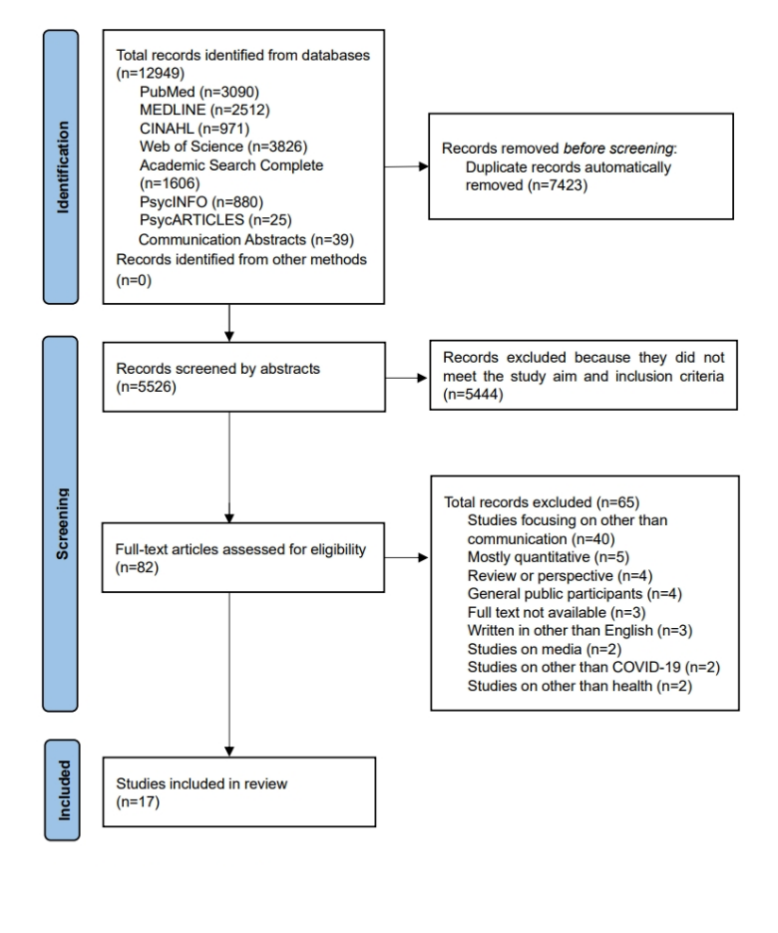
PRISMA (Preferred Reporting Items for Systematic Reviews and Meta-Analyses) search process flowchart.

Figure 1 shows the PRISMA flow diagram of the study selection. We included 17 studies in this review. Table 1 shows the characteristics of the included studies. A total of 5 studies were conducted in the United States, 4 in Canada, 2 in Switzerland, and 2 in Iran, and the other studies included participants from Europe, the Middle East, Asia, South America, and Africa. Participants’ occupations included communication specialists, medical professionals, scientists, and officials in public health institutions and local municipalities. The median number of study participants was 20 (IQR 12.5-26), and 367 health professionals were represented overall. The time frame in which the data were collected was from March 2020 to December 2022. The included studies showed an overall good methodological quality; the median number of studies classified as “yes” was 8 (IQR 7-9). Results of the quality appraisal are shown in Multimedia Appendix 1 [11,15,37-51].

**Table 1:** Study characteristics of qualitative studies regarding crisis communication during the COVID-19 pandemic.

Study	Country	Settings	Participants, n	Period when the data were collected	Study aim
Atighechian et al [15], 2021	Iran	Universities, governments, and hospitals	Health professionals and experts, including university faculty members, policy makers, physicians, and nurses working in the infectious disease unit (n=19)	March 2020-June 2020	To identify the challenges of COVID-19‑related information among people in point of experts’ views
Nehushtan et al [41], 2023	Israel	14 municipalities	Officials in local municipalities, including chief executive officers, mayors, and officials responsible for health in emergencies (n=42)	October 2020-February 2021	To explore local municipalities’ management of the COVID-19 pandemic
Sears et al [43], 2024	United States	One state	Public health workers, including sanitarian, educator, and administrative positions (n=11)	October 2020-March 2021	To gain an in-depth perspective of public health workers’ experiences during the complex and dynamic climate the COVID-19 pandemic
Colman et al [37], 2021	Belgium, the Netherlands, United Kingdom, Sweden, and Germany	Academic or public health research institutions	Scientists with an official government advisory role during the pandemic (n=21)	December 2020-April 2021	To explore the views and experiences of scientists working on government advisory boards
Bravo et al [40], 2023	Paraguay, Uruguay, United States, Canada, Germany, Spain, New Zealand, Australia, and South Korea	Universities, governments, a research center, a health care center, and a nongovernmental organization	Experts with experience in health crisis management or risk communication (n=10)	December 2020-March 2021	To identify a framework for risk communication during health crises from the voices of international experts by using the COVID-19 pandemic as a case study
Rubinelli et al [11], 2023	Switzerland	Institutions responsible for communicating with the public at the national and cantonal levels	Individuals responsible for public institutional communication within key public health institutions (n=25)	January 2021-July 2021	To collect individual experiences of communicating the situation and protective measures to the public
Ort and Rohrbach [42], 2024	Switzerland	Key Swiss public health institutions at the federal and cantonal levels	Individuals responsible for public institutional communication within key public health institutions (n=25)	January 2021-July 2021	To explore public health institutions’ challenges in implementing their COVID-19–related communication strategies
Pringle et al [50], 2022	Canada	Vancouver, the most diverse area	Communication specialists, medical professionals, officials in community service organizations, and volunteer community advocates (n=27)	May 2021-November 2021	To examine how community leaders, advocates, and public health communication specialists have approached community engagement
Engdawork et al [45], 2024	Ethiopia	A capital city	Stakeholders in the local government and private sectors engaged in social interventions to prevent COVID-19 (n=21)	September 2021-October 2021	To investigate the effectiveness of structural interventions during the earlier period of the pandemic in promoting adoption of preventive actions, challenges encountered during implementation, and draw lessons for future pandemic responses in low- and middle-income settings
Dubé et al [38], 2022	Canada	One province	Communication specialists in charge of developing health authorities’ COVID-19 communication and health care professionals actively engaged in public discussion in traditional and social media (n=11)	September 2021-December 2021	To explore how communication specialists working in health and governmental institutions and health care professionals have communicated about COVID-19
Lowe et al [39], 2022	Canada	11 jurisdictions	Public health officials, frontline health care workers, health scholars (social, epidemiological, policy, and clinical researchers), and health care worker union leaders (n=34)	September 2021-December 2021	To assess COVID-19 pandemic public health messaging for its potential to encourage or undermine public trust and adherence
Ittefaq [44], 2023	United States	Three states	Communication officials working in local health departments (n=14)	February 2022-April 2022	To explore challenges in information dissemination on social media, and factors contributing to burnout among communication officials
Kamruzzaman et al [49], 2023	Bangladesh	Three divisions that reported the highest COVID-19 cases	Health professionals, including district-level health education officers, residential medical officers, and pertinent national specialists (n=14)	February 2022-May 2022	To understand how the social context influences risk communication and community response during the COVID-19 pandemic
Bates et al [46], 2023	United States	One county	Public health professionals working at city health departments and a county health department (n=7)	March 2022-May 2022	To determine how public health officials perceived misinformation and political polarization during the pandemic, and to learn more about strategies county health officials used to combat misinformation
Strand et al [48], 2023	United States	Midwestern states	Public health professionals in local and state public health departments, universities, and health care organizations (n=48)	Summer of 2022	To describe the lived experiences of public health professionals working during the COVID-19 pandemic and to provide lessons learned and best practices to inform preparation for a future infectious disease pandemic
Bazrafshan et al [47], 2023	Iran	Provincial and national public health institutions	Public health professionals across provincial and national health authorities (n=20)	October 2022-December 2022	To develop a conceptual framework for health risk communication and infodemic management during epidemics and health emergencies
Johnston et al [51], 2024	South Africa and Zambia	18 community health organizations	Individuals working in community health organizations with engagement in health education and information services (n=18)	Not mentioned	To investigate the strategies, challenges, and needs of community health organizations involved in public COVID-19 education to understand their role in public health crises in relation to communicating health information

### Data Synthesis

Our analysis identified 242 free codes, which were organized into 41 descriptive themes: 7 analytical themes and 20 subthemes. Multimedia Appendix 2 [11,15,37-51] shows the analytical themes and subthemes, the studies that contributed to those themes, and direct quotations from the included studies to support those themes.

### Difficulties in Pandemic Communication

#### Gap Between Scientific Uncertainty and Expectations of Certainty

The COVID-19 pandemic revealed a gap between the normal reality of scientific uncertainty and political and public expectations of certainty, which made public health communication difficult [11,37-40]. The traditional scientific method of generating, evaluating, and acting on evidence was incompatible with the urgency of the pandemic [11,37]. However, participants were required by policy makers and citizens to provide rapid, definitive conclusions and explanations based on uncertain evidence in an uncertain situation [11,37,38] (quotation 1). This demand contrasted with the “slowness” of science [37]. Changes were unfolding rapidly in terms of scientific knowledge, the spread of the infection, and political, economic, and social conditions, and this required several changes in public health policies over a short time [37,38] (quotation 2). The gap between the uncertainty of science and unrealistic expectations of certainty resulted in public criticism of public health professionals and difficulties in public health communication [11,37-40] (quotation 3).

#### Communication Challenges in a “Slow Disaster”

Participants described the characteristics of the COVID-19 pandemic as a “slow disaster” [40]. Most disasters are short-lived, but the nature of the COVID-19 pandemic meant that they had to continuously deal with changing circumstances [11,40,41]. In the early stages of the pandemic, citizens cooperated with public health recommendations [11]. However, over time, their patience waned, their trust in public health professionals declined, and compliance worsened [11,40,41] (quotation 4). In addition, health professionals experienced difficulty in using communication to encourage citizens to adopt preventive behaviors amid fatigue from a pandemic with no seeming end in sight [11,40-42] (quotation 5).

### Difficulties Caused by the Infodemic

#### Difficulties in Public Health Activities Due to Misinformation

Misinformation about the severity and mortality of COVID-19 and the safety of vaccines spread on social media, and affected citizens’ attitudes and behaviors [11,15,42-47] (quotations 6 and 7). Participants were forced to devote significant resources to identifying and correcting misinformation [11,15] (quotations 8 and 9) but did not have effective measures to counter the sensational communication strategies used by purveyors of misinformation [11,39,43] (quotation 10). It was also more difficult to persuade people who had acquired a skeptical attitude through misinformation than it was to simply convey correct information [11,42,43,45].

#### Countering Misinformation

During the COVID-19 pandemic, participants learned several strategies to deal with misinformation. The first was the importance of timely communication; it was crucial that participants disseminated messages before misinformation spread [11,15,47] (quotation 11). Second, participants recognized that social listening improved their understanding of citizens’ psychosocial aspects and information needs, as well as the quality of information they provided [11] (quotations 12 and 13). Third, participants had to recognize and address fear and anxiety among citizens [39] (quotation 14). Finally, participants recognized the importance of actively using social media to disseminate accurate information and guide people to reliable information sources [11,38,39,46,47] (quotation 15). However, the lack of human resources with expertise in using social media made it difficult to counter misinformation using these platforms [11] (quotation 16).

### Difficulties in Partnerships Within and Outside Public Health

#### Tensions Within the Community of Public Health Experts

Participants recognized the importance of public health agencies partnering with epidemiologists, data scientists, sociologists, communication scholars, and other professionals with unique expertise for developing and implementing pandemic communication strategies [11,15,37,38,40,42,47-49]. This was because pandemic communication had to incorporate consideration of the social, economic, and political context that unfolded along with the health crisis [15,37,40,47-49] (quotation 17). However, there were coordination difficulties, especially in the early stages of the pandemic. Expert committees tended to be dominated by biomedical and virological researchers and often excluded sociologists and anthropologists [37] (quotation 18). A reason cited for the limited effectiveness of communication to citizens was that the strategies used lacked an understanding of people’s sociocultural beliefs [38,49].

#### Tensions Between Public Health and Politics

The conflict of interest between health care and the economy was a major factor that characterized the communication difficulties during the COVID-19 pandemic [11,15,37-39,42,43,46,48,49] (quotation 19). The conflict between safeguarding public health and maintaining the economy abrogated the coherence of policy decisions and messages to the public and led to public confusion and distrust of public health [15,37-39,48]. This conflict of interest between health care and the economy also created tensions between public health professionals and political leaders who wanted to maintain their political popularity [37,48]. At the policy-making level, some political leaders did not accept or use the scientific evidence provided by public health experts [37,39,48,49]. Moreover, political leaders sometimes used and abused public health professionals to evade their own responsibilities in communicating with the public [37,46] (quotation 20). At the policy practice level, public health professionals were sometimes obstructed by political leaders from recommending preventive behaviors and vaccination for citizens, rather than receiving political support [43,46] (quotations 21 and 22).

#### Difficulties in Coordination Between Public Health and Mass Media

Participants recognized the importance of close collaboration with the mass media [11,15,37,42,43,47]. They understood the influence of mass media in shaping public opinion and journalists’ commitment to scientifically accurate and balanced reporting [15,37,47]. However, they recognized that during the COVID-19 pandemic, the mass media often engaged in misleading reporting, as well as pitting public health professionals against each other and politicians against public health professionals, quoting out of context, and linking public health professionals to specific political decisions [11,37,42,43]. In addition, some participants perceived that the biased discussion and criticism of public health activities in mass media coverage led to a decline in people’s trust in public health [43] (quotations 23 and 24).

### Difficulties in Community Engagement

#### Need to Tailor Communication to Community Realities

Some participants recognized that many of the COVID-19 recommendations were not consistent with community realities [45,50,51]. For example, small living quarters, large families, and essential travel by public transportation to buy food and work affected compliance with preventive behaviors such as social distancing [45,51]. Some citizens had to prioritize other essential living activities over infection prevention behaviors. For example, people of lower economic status had to go out to earn their living even during lockdown periods [45,51] (quotations 25 and 26). Compliance with COVID-19 prevention recommendations meant that many citizens faced economic hardship, food insecurity, domestic violence, and mental health problems [45].

#### Need to Consider Local Cultural Factors

Cultural factors such as a given community’s dominant religion could also pose a barrier to compliance with the COVID-19 prevention recommendations [38,39,45,50] (quotations 27 and 28). However, participants recognized that cultural factors could act as both facilitators and inhibitors of public health activities [50]. They adapted their communication strategies to reflect community-specific sociocultural factors and incorporated ideas such as using culturally significant meeting places (eg, local religious centers) [45,50] (quotations 29 and 30).

#### Need for Bottom-Up and 2-Way Communication

Participants identified that the effectiveness of communication from health professionals to communities was inhibited by its 1-way nature [45,47,49,51] (quotation 31). Community groups and leaders were involved in implementing infection prevention programs; however, they had little involvement in planning and designing feasible programs [45]. Community participation tended to be lower when information was distributed from public health agencies to communities in a top-down manner. These top-down communication strategies, which lacked collaboration with the community, inhibited acceptance of recommendations for preventive behaviors [45,47,49]. This suggested that bottom-up and 2-way communication that involved the community were required to foster community engagement [45,47,49] (quotation 32).

#### Need to Build Trust With Communities

Participants generally responded that a trusting relationship between public health and the community was a factor in increasing community engagement [39,40,45,46,48,50,51]. They noted the importance of building trust with local political, religious, business, and agricultural leaders, along with schools, newspapers, radio stations, and other local organizations [40,48,50,51] (quotations 33 and 34). Existing local networks were especially important in developing grassroots communication activities [46] (quotation 35). In addition to trusting relationships with organizations, participants stated the importance of one-on-one trust relationships between health professionals and local residents [39,46] (quotation 36). However, in areas where public health outreach services had been reduced in the years before the pandemic, it was difficult to quickly rebuild trust between public health professionals and the community during the pandemic [50].

#### Need for Communication Through Community Channels

Communication through community-specific communication channels, such as local television and radio stations, social media platforms, and connections with trusted individuals, were emphasized as ways to increase community engagement [11,38,40,41,44-46,49-51] (quotation 37). Formal and informal communications were developed, including traditional media campaigns and disseminating messages via social media [11,40,41,45,50,51] (quotation 38). Participants noted that the key communication channels, including newspapers, radio, and social media, varied by community resident group [11,40,44,45,50,51] (quotation 39). For those groups using social media in particular, attempts were made to increase their engagement by encouraging their participation in communication activities [45,46] (quotation 40).

### Difficulties in Effective Communication

#### Need for Uniformity and Promptness in Communication

Participants identified the absence of reliable sources of information known to citizens as an impediment to effective communication [11,15] (quotation 41). The plethora of available information sources, including mass media and social media, created confusion among citizens [11,15,43] (quotation 42). Furthermore, the importance of rapid information dissemination was crucial in communication regarding a hitherto unknown infectious disease [11,15,39,44,47] (quotations 43 and 44). However, participants faced a dilemma whereby prioritizing the speed of communication did not allow sufficient time to create effective messages. For example, translation into multiple languages was time-consuming [11,44] (quotations 45 and 46). In addition, it took time to crunch the vast amount of information and create concise, clear messages [11,44,45] (quotation 47).

#### Need for Understandable and Persuasive Communication

Participants emphasized the importance of efforts to ensure the public understood messages [11,37-40,42,45,48] (quotation 48). These messages needed to have a clear purpose, use plain language and illustrations, and be persuasive to be easily understood and accepted by all citizens [11,38-40,45,48]. However, participants experienced difficulties in creating messages that addressed the various levels of citizens’ individual health literacy [38,39,42,45] (quotation 49). Understandable communication was also important for politicians and policy makers who did not necessarily have basic scientific knowledge [37].

#### Need for Communication to Empower People

Participants noted the harms associated with health authorities generating stigma for certain populations [38-40]. For example, they accused young adults of often failing to follow recommendations for social distancing, and therefore, transmitting the virus, or of prolonging the pandemic by not being vaccinated [38,39] (quotation 50). They stressed that effective communication strategies should emphasize helping people make better informed decisions rather than punishing them with blame or fear or offering temporary reassurance [38,40] (quotations 51 and 52).

### Burnout Among Communicators

#### Difficulties With Information Overload and Requests

Participants indicated that they felt like they were drowning in an overwhelming influx of information related to COVID-19 [11,41,51]. They tried to extract relevant information from this torrent; however, they did not know how to do so [51] (quotation 53). In addition, they were under intense pressure from the community to share the latest information about the novel virus [11,44] (quotation 54). Furthermore, public health professionals were expected to respond to constant media requests for updated information [11] (quotation 55).

#### Lack of Trust in Public Health

Participants experienced a lack of public trust, which led to communication difficulties [11,15,39,40,44,48,51]. A major contributing factor to this was discrepancies in the information disseminated by the government, municipalities, public health agencies, and professionals [15,39,48] (quotations 56 and 57). The confusion caused by these discrepancies increased people’s distrust and decreased their willingness to accept infection prevention recommendations [15,40,44] (quotations 58 and 59).

#### Attacks on Public Health Professionals by Citizens

Participants experienced criticism and attacks from citizens despite their best efforts to overcome the aforementioned difficulties [11,37,43,44,46,48] (quotation 60). Daily criticism and attacks from citizens through social media, email, and community face-to-face meetings accelerated burnout among participants [11,43,44,46,48] (quotations 61 and 62). Accordingly, they sought ways to prevent burnout, including learning to set emotional boundaries for criticism [43] (quotation 63). They noted that rare words of gratitude from citizens empowered them [37,43] (quotation 64).

### Need to Train Communication Specialists and Establish a Permanent Organization

#### Need to Train Communication Specialists

There was a notable lack of human resources with communication expertise during the COVID-19 pandemic [11,42,44,45,47]. In the early stages of the pandemic, public health agencies made efforts to increase the number of communications personnel by reorganizing their human resources [11]. However, securing a sufficient number of communications personnel, relative to the overwhelming volume of information that needed to be addressed, was difficult [11,42,45,47] (quotation 65). Personnel who had been moved to communications duties from other departments often lacked basic communication skills and competencies [11,42,45]. In addition, even those who had been previously trained in communications lacked the experience and ability to communicate effectively in the emergency pandemic situation [11,42] (quotation 66).

#### Need to Establish a Permanent Organization Specializing in Communication

Participants identified the rigidity within existing organizational structures as a problem. They emphasized that the many procedures, time-consuming approval processes, and inflexible and rigid protocols in the organizations inhibited rapid and effective public health communication [11,50]. They agreed on the importance of establishing a permanent organization specializing in public health communication [40,41] and noted that such an organization should train communication specialists, accumulate methods for effective communication strategies, build cross-functional partnerships with other organizations, and establish a structure to respond quickly in further public health crises [47] (quotation 67).

### Conceptual Model

**Figure 2:**
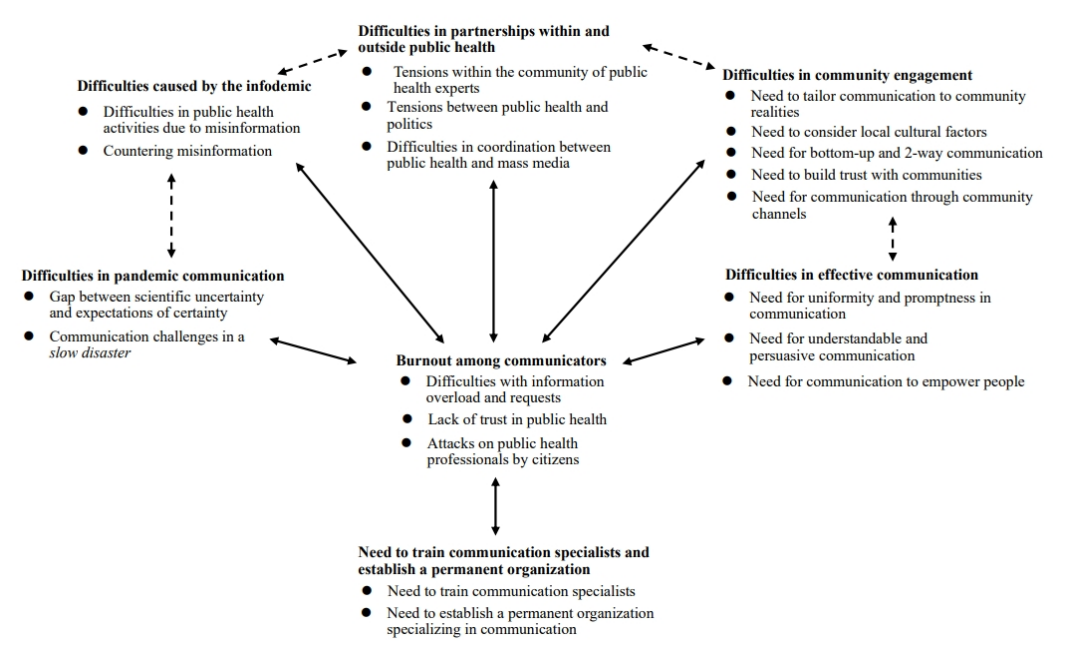
Conceptual model developed from qualitative studies regarding crisis communication during the COVID-19 pandemic.

Figure 2 shows the conceptual model developed from the above results. Themes 3.2.1 to 3.2.5 were interrelated, and the difficulties experienced by communicators resulted in their burnout (theme 3.2.6). The difficulties and needs indicated in themes 3.2.1 to 3.2.6 indicated the need for future training of communication specialists and establishing a permanent organization specializing in communication (theme 3.2.7). It was assumed that training experts and establishing organizations would reduce difficulties and enable effective communication in subsequent public health crises.

## Discussion

### Principal Findings

This systematic review of qualitative studies examined the difficulties, challenges, and needs experienced by public health professionals during the COVID-19 pandemic and identified 7 themes. The theme of difficulties in pandemic communication encompassed difficulties stemming from scientific uncertainty and the “slow disaster.” Public health crisis communication inherently involves uncertainty [52,53], and the WHO and the Centers for Disease Control and Prevention (CDC) recommended explicitly communicating information about uncertainties [1,54,55]. Researchers in crisis communication argued that communicating uncertainty increased rather than decreased public trust [56,57]. However, this systematic review revealed that risk communication in the real world is not as simple as the above recommendation suggests. Uncertainty reduction theory indicates that humans are intrinsically motivated to reduce uncertainty [58]. Therefore, communicating uncertainty creates a conflict with people’s demand for certainty. However, when people’s trust in their government and communicators is stronger, they tend to more successfully accept uncertainty [59]. People’s trust in government and public health agencies may offer a clue to resolving communication difficulties associated with uncertainty. Furthermore, neither the WHO nor CDC guidelines contained details on how to deal with communication difficulties stemming from a slow disaster [1,54,55]. Coping with pandemic fatigue was one of the difficulties stemming from the slow disaster. Although previous studies have examined factors associated with pandemic fatigue during the COVID-19 pandemic [60,61], much remains unknown about pandemic fatigue. Further research should consider effective communication strategies for a slow disaster.

The communication difficulties in the COVID-19 pandemic were characterized by the destructive impact of the infodemic. A survey conducted in the United Kingdom in 2020 showed that 46% of the public had been exposed to fake news about COVID-19 and 40% said they could not tell the difference between truth and lies [62]. Previous studies have examined effective debunking methods for misinformation [18-21]. The CDC also developed public health infodemic surveillance systems in the wake of the COVID-19 pandemic [63]. Furthermore, there are more than 100 laws against disseminating misinformation in different countries worldwide [64]. A multifaceted approach is needed to prepare for future public health infodemics, including surveillance, communication, and legal regulation.

The WHO guidelines to address COVID-19 emphasized the importance of collaboration within public health agencies and with external partners [55]. However, this systematic review found that, in reality, tensions in and outside of public health agencies hindered an effective crisis response. During noncrisis periods, governments, public health agencies, researchers, and media are often siloed, making crisis-related coordination and information sharing difficult [65]. In addition, political and economic interests that conflict with public health policies hinder an effective pandemic response [66]. Such partnership failures, which were experienced in past epidemics and pandemics, were repeated in the COVID-19 pandemic. Addressing this is a crucial challenge going forward.

Existing public health organization guidelines emphasized the importance of community engagement strategies [1,54,55]. Many studies have shown that community-based cultural factors were related to preventive behaviors and mortality rates during the COVID-19 pandemic [67-70]. Furthermore, language and cultural barriers prevented access to information, understanding of messages, and compliance with recommendations during the pandemic [71,72]. This systematic review showed that top-down, 1-way communication to the community hindered effective pandemic responses, despite the importance of a bottom-up approach that involves community stakeholders and residents in decision-making having been officially emphasized [73]. Communication in public health crises requires adapting communication strategies to the cultural, social, and demographic background of the local community to gain support among the target population [74]. To achieve this, it is important to break away from top-down, 1-way communication and adopt a 2-way, bottom-up approach that includes dialogue with the community [75].

During epidemics and pandemics, it is important that information from public health agencies is not overtaken by competing misinformation [25]. The first message that an audience receives shapes their subsequent attitudes [76]. Therefore, quick dissemination of information based on partial evidence is better than delayed dissemination of information based on complete evidence [1,55,77] because prompt communication is an essential principle of risk communication [54]. However, this systematic review revealed that the speed of communication hindered the effectiveness of communication during the COVID-19 pandemic. Public health professionals experienced difficulty in securing time for translation, pretesting, and creating easy-to-understand messages as they were under pressure to communicate quickly. The COVID-19 pandemic highlighted the difficulty of following existing crisis communication guidelines in a real-world crisis response.

Many public health professionals experienced burnout during the course of the pandemic. The main factors contributing to burnout were information overload that exceeded limited human resources, along with criticism and attacks on public health professionals from the public. The lack of public trust in public health also contributed to attacks against health professionals. The degree of trust in public institutions was associated with the rate of COVID-19 infection and the associated mortality rate [78]. A 2022 report by the Organisation for Economic Co-operation and Development highlighted that public trust was a key insight from the evaluation of responses to the pandemic, which pointed to the importance of building trust over a long period before a crisis occurs [73]. Building public trust and preventing burnout among public health professionals are essential for preparing for future public health crises.

The aforementioned 6 themes suggested the seventh theme, the need to train communication specialists and establish permanent organizations specializing in communication. These measures are necessary to address the aforementioned issues brought to light by the COVID-19 pandemic. COVID-19 showed that many countries had failed to learn the lessons of past global infections (eg, severe acute respiratory syndrome and influenza A virus subtype) and had failed to prepare for a future public health crisis [73,79]. Even now, many countries are still not prepared for future public health crises [80]. Another public health crisis occurring is not a matter of “if” but of “when” [81]. The best way to manage a crisis is to prevent one [25], and the second-best way to manage a crisis is to prepare for one [82]. All public health institutions and professionals must learn from the difficulties, challenges, and needs identified in this systematic review and update their strategies and guidelines to implement more effective communication in the next public health crisis.

### Future Directions for Practitioners

The results of this systematic review suggest the following practice implications, which may help to prepare for the next public health crisis. (1) The scientific process is accompanied by uncertainty; however, politicians and citizens seek certainty. It is necessary to increase trust in public health organizations and address the communication difficulties associated with uncertainty, to address pandemic fatigue, and to develop effective communication strategies for future slow-onset disasters. (2) More research and practice are needed to manage misinformation in public health crises, including surveillance and communication strategies for “prebunking” and debunking information. (3) Partnerships between stakeholders at both the policy-making and communication practice levels are needed to manage public health crises. Such partnerships are important for enabling the creation and transmission of consistent messages, and avoiding confusion among citizens and distrust in public health. (4) It is necessary to build trusting relationships between public health organizations and communities before a crisis occurs and to enable bottom-up communication during crises. (5) It is also necessary to address the trade-off between communication promptness and effectiveness and conduct communication with the aim of empowerment. (6) Measures are needed to prevent burnout among health professionals during a crisis. (7) To address these issues and support an effective response to future public health crises, it is necessary to train more communications specialists, establish permanent organizations specializing in communication, and update strategies and guidelines.

### Limitations

This systematic review had several limitations. First, we conducted a rigorous literature search and qualitative synthesis with 2 or more reviewers. However, we could not completely rule out the possibility that some relevant literature had not been included. Second, we did not weight the interpretation of study results according to the quality appraisal of the included studies; however, the included studies showed an overall good methodological quality. Third, because this was a systematic review of previous studies, our interpretations were limited by the data that were reported in the included studies. Fourth, participants in the included studies had various occupational backgrounds such as policy makers, officials in local municipalities, frontline health care workers, and scientists. A strength of this review was that it reflected the experiences of participants from diverse backgrounds; however, it was also limited by not differentiating experiences at the policy-making level from those at the policy implementation level on the front line. Fifth, another strength was that we included studies from various countries in Europe, the Middle East, Asia, Africa, and North and South America; however, a limitation was that we did not make any economic or cultural distinctions. Finally, because all crises are novel and involve contextual differences, the generalizability of the findings and implications of this study to future crises is limited. Despite these limitations, this review has the important implications mentioned earlier, in that it identified the gaps between existing crisis communication guidelines and real-world crisis communication and the difficulties and needs that arise from those gaps.

### Conclusions

This systematic review of qualitative studies identified the following issues that need to be addressed to prepare for subsequent public health crises. Despite the importance of collaboration within and outside public health and community engagement being highlighted in existing crisis communication guidelines, there was insufficient preparation and response to the COVID-19 pandemic. Although prompt communication is an essential principle for crisis response, the trade-off between promptness and the effectiveness of communication should be addressed. Difficulties specific to “slow disasters” and “infodemics” characterized the challenges encountered during the COVID-19 pandemic. Information overload, a shortage of human resources, and a lack of trust in public health contributed to burnout among health professionals. Public health professionals need to address the difficulties and needs identified in this systematic review by training communication specialists and establishing permanent organizations specializing in communication. One health professional described the difficulties resulting from the lack of preparation during the COVID-19 pandemic as “we are building the plane while we are flying” [44]. Of course, airplanes must be built before they fly, and in the case of a public health crisis, preparations must be made before the crisis arises.

## Appendix

Multimedia Appendix 1 Quality appraisal of included studies.

Multimedia Appendix 2 Themes and illustrative quotes from qualitative studies regarding crisis communication during the COVID-19 pandemic.

Multimedia Appendix 3 PRISMA (Preferred Reporting Items for Systematic Reviews and Meta-Analyses) checklist.

## Abbreviations

CDC: Centers for Disease Control and Prevention
PRISMA: Preferred Reporting Items for Systematic Reviews and Meta-Analyses
WHO: World Health Organization

## Data Availability

Data sharing is not applicable to this paper as no datasets were generated or analyzed in this study protocol.
