# Supplementary material for: Experiences of Public Health Professionals Regarding Crisis Communication During the COVID-19 Pandemic: Systematic Review of Qualitative Studies
Source: JMIR Infodemiology. 2025 Mar 14;5:e66524. doi: 10.2196/66524 (PMC11953600; doi:10.2196/66524)
Supplement: Multimedia Appendix 1 [file infodemiology_v5i1e66524_app1.doc]

| **Appendix 1. Results of the quality appraisal of included studies according to the JBI critical appraisal checklist for qualitative research** | | | | | | | | | | | |
| --- | --- | --- | --- | --- | --- | --- | --- | --- | --- | --- | --- |
| Study | 1 | 2 | 3 | 4 | 5 | 6 | 7 | 8 | 9 | 10 | Number of Yes |
| Atighechian et al. 2021 [15] | No | Yes | Yes | Yes | Unclear | No | Yes | Yes | Yes | Yes | 7 |
| Nehushtan et al. 2023 [41] | Unclear | Yes | Yes | Yes | Unclear | Unclear | Unclear | Yes | Yes | Yes | 6 |
| Sears et al. 2024 [43] | Yes | Yes | Yes | Yes | Yes | Yes | Yes | Yes | No | Yes | 9 |
| Colman et al. 2021 [37] | Unclear | Yes | Yes | Yes | Yes | Unclear | Yes | Yes | Yes | Yes | 8 |
| Bravo et al. 2023 [40] | Unclear | Yes | Yes | Yes | Yes | No | Yes | Yes | Yes | Yes | 8 |
| Rubinelli et al. 2023 [11] | Yes | Yes | Yes | Yes | Yes | No | Yes | Yes | Yes | Yes | 9 |
| Ort and Rohrbach 2024 [42] | Unclear | Yes | Yes | Yes | Yes | No | Yes | No | Yes | Yes | 7 |
| Pringle et al. 2022 [50] | Yes | Yes | Yes | Yes | Yes | Unclear | Yes | Yes | Yes | Yes | 9 |
| Engdawork et al. 2024 [45] | Yes | Unclear | Yes | Yes | Yes | Yes | Yes | Yes | Unclear | Yes | 8 |
| Dubé et al. 2022 [38] | Yes | Yes | Yes | Yes | Yes | Unclear | Yes | Yes | Yes | Yes | 9 |
| Lowe et al. 2022 [39] | Yes | Yes | Yes | Unclear | Yes | No | Yes | Unclear | Yes | Yes | 7 |
| Ittefaq 2023 [44] | Unclear | Yes | Yes | Yes | Yes | No | Yes | Yes | Yes | Yes | 8 |
| Kamruzzaman et al. 2024 [49] | Unclear | Yes | Yes | Yes | Yes | Yes | Yes | Yes | Yes | Yes | 9 |
| Bates et al. 2023 [46] | Unclear | Yes | Yes | Yes | Unclear | No | Yes | Yes | Yes | Yes | 7 |
| Strand et al. 2023 [48] | Unclear | Yes | Yes | Yes | Yes | No | Yes | Unclear | Yes | Yes | 7 |
| Bazrafshan et al. 2023 [47] | Yes | Yes | Yes | Yes | Yes | Unclear | Yes | Unclear | Yes | Yes | 8 |
| Johnston et al. 2023 [51] | Unclear | Yes | Yes | Yes | Yes | Unclear | Unclear | Yes | Yes | Yes | 7 |
