# Supplementary material for: Experiences of Public Health Professionals Regarding Crisis Communication During the COVID-19 Pandemic: Systematic Review of Qualitative Studies
Source: JMIR Infodemiology. 2025 Mar 14;5:e66524. doi: 10.2196/66524 (PMC11953600; doi:10.2196/66524)
Supplement: Multimedia Appendix 2 [file infodemiology_v5i1e66524_app2.docx]

**Multimedia Appendix 2.** Themes and illustrative quotes from qualitative studies regarding crisis communication during the COVID-19 pandemic.

| Themes | | Studies contributing to themes | | Illustrative quotes |
| --- | --- | --- | --- | --- |
| **Difficulties in pandemic communication** | | | | |
|  | The gap between scientific uncertainty and expectations of certainty | [11,37-40] | - Quotation 1: “You actually have to think very quickly, react very quickly and also develop your opinion very quickly, which of course contrasts with the slowness of science.” [37] - Quotation 2: “If you have results that are two weeks old, they are already a bit outdated and of limited use.” [37] - Quotation 3: “Suddenly scientific processes took place in public, scientific results or results of studies were uploaded on preprint servers and then discussed in public - even the collegial discussion took place in public, instead of in the academic setting as before, where it was only published when there was agreement. That was something that the public, the Swiss public, couldn’t deal with in the beginning. They didn’t speak the same language. Science said one thing and the population understood it differently. That is my personal view of things. That made it difficult to communicate.” [11] | |
|  | Communication challenges in a “slow disaster” | [11,40-42] | | - Quotation 4: “In the second wave, the situation was very different, there was also talk of pandemic fatigue, and press conferences no longer played such a decisive role.” [11] - Quotation 5: “I mean, ultimately, a classic crisis communication, now if a plane crashes or a creek floods, you know roughly what’s important. But after three or four days, it’s done, or after two weeks, and then you’ve gone through the whole thing, and you can recover. And in that case, I think in classic crisis communication, you could quickly check everything off. Communicate constantly, repeating, announcing when you’re going to communicate next time, etc. But over such a long distance, I don’t think anyone really had a clue how to drive the communication strategy for a year or even longer. And we didn’t have that expertise either.” [11] |
| **Difficulties caused by the infodemic** | | | | |
|  | Difficulties in public health activities due to misinformation | [11,15,39,42-47] | | - Quotation 6: “We have a lot of people that don’t view [the virus] as real... People just don’t want to hear the truth.” [43] - Quotation 7: “Many people considered the pandemic as a doom’s day evil with no solution, and many others also believed vaccines to be microchips maliciously distributed by western countries. This created vaccine hesitancy and skepticism towards other preventive measures.” [45] - Quotation 8: “I spend almost 90% of my time dealing with and denying false news.” [15] - Quotation 9: “On social networks, it seems to me that there were phases, especially in the beginning, where we said ‘OK, every time there is false information, we’ll correct it.’ And then afterward, there was so much that it was almost impossible to tell and to explain why it was false.” [11] - Quotation 10: “Anti-vaxxers are using fear tactics and they don’t have to say things that are true. But they can say things that are scary or completely biased. And so, it’s not a fair game. I think we need to beef up our capacity to deal with things like that.” [39] |
|  | Countering misinformation | [11,15,38,39,46,47] | | - Quotation 11: “We were too slow off the mark. I think some of the antivaccine messages had already set in...And I think we could have messaged earlier.” [39] - Quotation 12: “We have had our own fake news media review for a few months now where we also look at what information there is that we might have to react to. We also had a summary of all the media coverage from an external source several times a week, who then gave his input, so that we also have an external view of the whole thing.” [11] - Quotation 13: “We have a media database where we get saved search results. We look at that every morning and discuss it in a team meeting and try to extract learnings from it...If our messages are getting through, or if we have to adjust it” [11] - Quotation 14: “You can’t just say, ‘That’s not true.’ Because that’s not emotionally compelling. It’s not persuasive...I think there have been some emotionally compelling things about ‘We’re all going to get back to doing things we love.’ And I think in some cases, that’s been effective.” [39] - Quotation 15: “People like to ‘do their research.’ And that can be a problem because they go to deep corners of the internet, and they get wrong information...And in providing them with correct information, coming up with different strategies but also connecting them with different pathways of information.” [39] - Quotation 16: “[We realized] that we would have needed a social media manager, a competence that we didn’t have in the group, it’s a specific competence that it’s not related to a designer, you can find one, it’s not related to a communicator, that is a different figure with different competences that in our way it was missing.” [11] |
| **Difficulties in partnerships within and outside public health** | | | | |
|  | Tensions within the community of public health experts | [11,15,37,38,40,42,47-49] | | - Quotation 17: “We have to build and understand the problem well, what is the problem we are facing as a society, what problems are all of the citizens facing, to understand that there would be a scientific-expert, political-citizen, public and transparent debate, in order to build the ways by which it has to be addressed.” [40] - Quotation 18: (talking about interdisciplinary collaboration) “That was most frustrating because we were also sitting at the table with people who didn’t want to understand. You should at least be open to the vision of one another.” [37] |
|  | Tensions between public health and politics | [11,15,37-39,42,43,46, 48,49] | | - Quotation 19: “We have a great tension between economic interests and health interests. The Director of Economic Affairs, of course wants to relax as little damage to the economy as possible, few restrictions and so on. And on the other side there is the Minister of Health. He is responsible for the health of the population… These are enormous areas of tension, the different interests that interact, and this is one example of many.” [11] - Quotation 20: “At some point in March, our Prime Minister said ‘I do what the [name of advisory board] says.’ I found that a very unpleasant comment, because it implies that you are directly responsible for the policy pursued and that is not the case. It is simply a political choice. And we only give advice. So they should take it as such and not hide behind that advice.” [37] - Quotation 21: “We don’t have the backing to enforce [the orders]. So, we rely on trying to convince people it’s the right thing to do. That’s basically been our method.” [43] - Quotation 21: “I cannot change the political leaders in the village next door to me that basically chastised the health officer in an open public forum and said that what they are doing is unnecessary and inappropriate.” [46] |
|  | Difficulties in coordination between public health and mass media | [11,15,37,42,43, 47] | | - Quotation 23: “There was an article published about my health department the other day...It was just such a negative article, and I’m like, ‘Man. We are trying so hard. You have no idea what the behind the scenes looks like.’ It’s kind of like a slap in the face.” [43] - Quotation 24: “The lack of understanding of what public health is kind of makes me crazy...When they talk about what a poor response it has been to this crisis, I get angry because they forget about all of us down here doing the work and busting our butts. You forget that we’re still here and we’re still doing it.” [43] |
| **Difficulties in community engagement** | | | | |
|  | Need to tailor communication to community’s realities | [45,50,51] | | - Quotation 25: “It’s easy to tell everyone to go and stay in their own homes, but for the kids on the streets...they rely on donations or begging to get food… there are some families as well that have had nothing.” [51] - Quotation 26: “Many poor people were out of their homes searching for opportunities to earn daily income for their survival giving a deaf ear to restrictions of movements, staying at home and social distancing.” [45] |
|  | Need to consider local cultural factors | [38,39,45,50] | | - Quotation 27: “The long-standing culture of practicing social events, compromised the public acceptance of preventive measures against the pandemic.” [45] - Quotation 28: “Many people were disregarding public health measures; seeking solutions to come only from their faith.” [45] - Quotation 29: “We’ve noticed that a lot of them are coming out to the ‘Inreach’ community clinics like at the gurdwaras, mandirs, and mosques, where they feel comfortable going... And we’ve heard from the seniors making comments like, ‘Okay, the vaccine is being given at a place of worship...We feel more safe in receiving the vaccine.’” [50] - Quotation 30: “We set up community-based information dissemination centers in each sub city. We worked with the association of iddirs and religious leaders to disseminate messages about the pandemic. This was effective because we were able to reach families with information on prevention measures at an early stage of the pandemic.” [45] |
|  | Need of bottom-up and 2-way communication | [45,47,49,51] | | - Quotation 31: “Risk communications and community engagement is not a one-way factor. Communication should be two-way. Like circulating information, the one-way communication was excellent but lacked community engagement.” [49] - Quotation 32: “Efforts should be made to involve the community to plan together in a bottom-up fashion of operation and participate in decision-making process as well.” [45] |
|  | Need to build trust with communities | [39,40,45,46,48,50,51] | | - Quotation 33: “Strong ties to community partners due to prior relationships and familiarity was very beneficial for rural communities, as we knew who to contact for resources.” [48] - Quotation 34: “It is extremely important to create strong collaborative relationships with community organizations in advance, because you will need to rely on those relationships in your response.” [48] - Quotation 35: “With our schools, with our elected officials here, with our common council, and our mayor, and our administration. Spending time at those meetings, sending regular updates, often by email earlier in the pandemic, or having phone calls with them to answer their questions and help them understand. Also, a lot of messaging to different groups of businesses. So, you know, to churches, to childcare centers, to restaurants and bars, other businesses.” [46] - Quotation 36: “We realized the one-on-one support was much more likely to lead to somebody then getting the vaccine if they were able to talk to a nurse or talk to somebody and answer those questions by somebody they trust, that was actually a medical person.” [46] |
|  | Need for communication through community channels | [11,38,40,41,44-46,49-51] | | - Quotation 37: “Social media is certainly more important. But you can still see that the older population goes to the website or informs itself via the newspaper or the free newspaper. Depending on the demographic and age group to some extent, we used different channels.” [11] - Quotation 38: “When [we] were doing home outreach, most people were actually surprised. They didn’t even know [about COVID-19]. They hadn’t even heard because they don’t have cell phones, they don’t have WhatsApp...most people needed to hear by word of mouth [which is] why we needed pamphlets.” [51] - Quotation 39: “South Asian communities—I’m talking about seniors in South Asian communities—They depend on radio. Okay? They are getting their information from radio, because they won’t go and search online. Yeah? They can’t do that.” [50] - Quotation 40: “As we look over the span of the pandemic to date, we really just want to recognize when we empowered grassroots community neighborhood members to amplify messages on their own social media platforms, Facebook, TikTok, you name it, whatever, that is where we really were seeing some of the most direct influence to some of the most vulnerable populations and had the best sort of reach.” [46] |
| **Difficulties in effective communication** | | | | |
|  | Need for uniformity and promptness in communication | [11,15,39,43-45,47] | | - Quotation 41: “People don’t know where to get information and which information source to trust.” [15] - Quotation 42: “People receive general information about COVID‑19 from various media outlets, but they do not have the same information about necessary actions, such as disinfecting surfaces. One source says make Javelle water and bleaching solution with a ratio of 1:4. Another source says make it with a ratio of 1:49, another says make it with the ratio of 1:100. Individuals and/or organizations give different instructions.” [15] - Quotation 43: “Due to the fact that the news and information about the Coronavirus unfortunately reached the people very late, the members of the community partially underestimated the epidemic, and no training was provided.” [15] - Quotation 44: “You have to at least give the impression that you are actively and quickly communicating and not holding anything back.” [11] - Quotation 46: “Having things in multilanguage is really challenging. So, having things translated, it takes a long time, and then someone has a question in a different language. Then, we have to translate the answer using online tools, so it might not be super accurate.” [44] - Quotation 46: “And that already shows that there are certain demands on time. We would have a media release and it might be urgent...But that can’t be sent out until the Romansh and Italian translation is also available. And that’s been a huge challenge in the whole pandemic story.” [11] - Quotation 47: “The general public is not going to understand that we are going to take the time to try to understand it. So, we need more of an hour, but preferably 4 hours. We can do it, then we may not have an effective message.” [44] |
|  | Need for understandable and persuasive communication | [11,37-40,42,45,48] | | - Quotation 48: “Science communication. The communication of studies, for example, the translation into a language that is understood and where the main points are really received and not what is actually only of interest to science, this is an ongoing process and something where you have to sit down with the experts and see how far we can break it down so that it’s understandable for the people outside.” [11] - Quotation 49: “Doctors and public health professionals are up here all the time, talking about efficacy. I had a client talk to me at one of the vaccine clinics, and she said, ‘I’m so glad to hear the vaccine has an 80% efficacy rate. It’s just such a shame that 20%of the people will die.’ Like, that was the way that the information was being read.” [39] |
|  | Need for communication to empower people | [38-40] | | - Quotation 50: “We’re really not giving people who have not been vaccinated a chance to feel good about getting vaccinated. We’ve already moved, like, fully into the shame mode, right? And that’s going to be hard for some people to walk back if they’ve got any kind of pride. So, you can try to force them with vaccine passports. But you know, they’re going to be resistant. So, we haven’t gotten that piece right.” [39] - Quotation 51: “I personally think that a fear-based appeal can only take people so far. It’s like the kind of decay effect that you get with graphic anti-drink driving ads where you see bodies and car parts strewn across the road, and after a while when these sorts of advertisements come on TV, people just change the channel.” [40] - Quotation 52: “I’m more interested in empowering [people] than reassuring them.” [38] |
| **Burnout of communicators** | | | | |
|  | Difficulties with information overload and requests | [11,41,44,51] | | - Quotation 53: “[The organization program manager] is basically drowning in information... [They] are really struggling because there’s just too much.” [51] - Quotation 54: “The first wave was characterized by thousands of mobile phone calls. Fortunately, I was given two telephones. One as the cantonal doctor and the other my private one, but everyone knew that too. And then they really did ring all the time, sometimes at the same time. Companies, old people’s homes, anyone who had a question, I was probably abused a bit as an information center.” [11] - Quotation 55: “Corona is so omnipresent everywhere in all the media. And the media people need information. I sometimes ask myself, what did the media do before Corona or what are they doing after Corona? I don’t know. But the need for information from the media is still extremely great.” [11] |
|  | Lack of trust in public health | [11,15,39,40,44,48,51] | | - Quotation 56: “Messaging and communication issues nationally and locally, that were confusing and not consistent.” [48] - Quotation 57: “The Ministry of Health and Medical Education and the health authorities do not have specific credible channels and entries, so weaknesses and conflicts are transferred to the community, then their authority is destroyed, and people lose confidence in official sources.” [15] - Quotation 58: “I think that...you know, the theories that we have in the best practices don’t pay enough attention to the fact that when you go into an emergency situation or a crisis situation...if the trust in those institutions is already low, you’re going to have to immediately deal with that before you can start thinking about the population accepting your proposed actions and solutions, right?” [40] - Quotation 59: “[In South Korea] the public trust in the government was more than like 80% or something. So, it was huge, a huge trust behind all the communication efforts. I think that was very helpful, and so the...you know, all those crisis communication...kinds of principles like fast, consistent, open and routine communication with a credible spokesperson is probably the key to this, you know, risk communication strategy.” [40] |
|  | Attacks on public health professionals from citizens | [11,37,43,44,46,48] | | - Quotation 60: “I was just really internalizing that stuff; being called idiots and all kinds of names from both sides. And that kind of caught me off guard. The people who didn’t think we were doing enough yelling and screaming...I never heard anything like that in my life. And then people on the other side, when we would put out education about how to stay safe or masks, yelling at us the other way. And I just thought, this is crazy. This is insanity day after day.” [43] - Quotation 61: “So, dealing with all the hate and anger we get on our page is just too much. It definitely affects you. [And] people are still so angry, or they are still questioning the vaccine and posting comments about it. It’s like, get a life—just move on. I’m tired of it.” [44] - Quotation 62: “Absolutely and I think it’s just been a thing throughout the entire pandemic...We’ve dealt with people not trusting us or them believing that we don’t have their best interest in mind. This definitely leads to burnout but [we’re] just kind of learning tools to fight through it.” [44] - Quotation 63: “I used to get really, really, really wrapped up in, you know...‘This is why social distancing, coupled with masks, coupled with hand washing, really works.’...It’s just so much easier to say, ‘We’re doing the best that we can with what we have...’ and go from there...And usually, people are kind of like, ‘Oh okay, she’s not going to argue with me,’ so they get a little quiet and the conversation ends, and we’re going about our day...You’re not going to change their mind. You’re not going to help them, because they don’t want your help...It is what it is.” [43] - Quotation 64: “You get yelled at, hung up on. People are so frustrated, and you get all of that. But then every now and then you’ll get that one person that’ll say, ‘Thank you for what you’re doing.’ That’s a good moment... Just one person saying, ‘Thank you for what you do.’ That’s kind of huge in this time.” [43] |
| **Need to train communication specialists and establish a permanent organization** | | | | |
|  | Need to train communication specialists | [11,42,44,45,47] | | - Quotation 65: “For us, it was clearly human resources first because when you have peaks of 4000 comments or 3000 comments in one day, you just need a human to be able to deal with them. The machine doesn’t do it. You need a bit of resources too. It’s still an important thing. That is clear.” [11] - Quotation 66: “This was kind of improvised at the beginning. So, we very quickly set up something of our own...so in the first wave, we had a communications hub that we staffed with internal people, but who actually did not have any training in communications per se...And it was kind of a mess.” [11] |
|  | Need to establish a permanent organization specializing in communication | [11,40,41,47,50] | | - Quotation 67: “Institutionalization is really helpful because if you have an office that is dedicated to pandemic communications, like South Korea and Taiwan, suddenly, when you need to start communicating with people, you just have the resources to be able to pump out: Facebook posts and tweets and memes and cartoons; and you can just do it! Because you have that capacity.” [40] |
